# NaCl exposure results in increased expression and processing of IL-1β in Meniere’s disease patients

**DOI:** 10.1038/s41598-022-08967-7

**Published:** 2022-03-23

**Authors:** Shresh Pathak, Andrea Vambutas

**Affiliations:** 1 The Feinstein Institutes for Medical Research, Manhasset, NY USA; 2 Department of Otolaryngology, The Apelian Cochlear Implant Center, Northwell Health System, New Hyde Park, NY USA; 3 Department of Otolaryngology, Donald and Barbara Zucker School of Medicine at Hofstra/Northwell, Hempstead, NY USA; 4 Department of Molecular Medicine, Donald and Barbara Zucker School of Medicine at Hofstra/Northwell, Hempstead, NY USA; 5 Department of Otorhinolaryngology - Head & Neck Surgery, Montefiore Medical Center, Albert Einstein College of Medicine, Bronx, NY USA

**Keywords:** Immunology, Medical research

## Abstract

Meniere’s disease (MD) is a chronic disease that causes episodic vertigo, fluctuating hearing loss, and aural fullness, initially managed by dietary salt reduction, and use of diuretics. Our prior research in autoimmune inner ear disease (AIED) demonstrated that in peripheral blood mononuclear cell (PBMC) from corticosteroid-resistant AIED patients, increased production, processing and release of interleukin-1β (IL-1β) is observed and hearing could be improved with use of anakinra, an interleukin-1 receptor antagonist. We have further identified that in these AIED patients, IL-1β is uniquely processed to a 28 kDa pro-inflammatory product by caspase-7. In the present study, we characterize the production, processing and release of the pro-inflammatory cytokines IL-1β and IL-6 from PBMC of MD (n = 14) patients in response to sodium chloride (NaCl), and determined the effect of the diuretic triamterene-hydrocholothiazide (T-HCTZ), or anakinra in these patients. We observed that PBMC cultured with NaCl from MD patients show processing of IL-1β to the 28 kDa product, and that this product is abrogated with T-HCTZ. Our observations are consistent with other autoimmune diseases where high concentrations of NaCl caused release of pro-inflammatory cytokines and may provide further insight as to the mechanism of disease progression in MD patients.

## Introduction

Meniere’s disease (MD) is disease resulting from endolymphatic hydrops in the cochlea which clinically causes fluctuating hearing loss, aural fullness^1^, tinnitus and episodic vertigo. Current criteria for MD were jointly formulated in 2015 by the Classification Committee of the Bárány Society and other scientific societies, including the Equilibrium Committee of the American Academy of Otolaryngology-Head and Neck Surgery (AAO-HNS). Only two categories are considered in this current classification: probable and definite cases of Meniere’s disease^2,3^. Initial management of MD is a low sodium diet and use of diuretics, although the evidence supporting their efficacy is limited^4–6^. In a meta-analysis of 50 years of studies from 11 countries, oral diuretic therapy may be advantageous in the medical management of Meniere’s disease, as diuretic therapy showed improvement in vertigo episodes but hearing improvement was inconsistent^7^. One of the initial double-blind cross-over placebo-controlled trials using Triamterene-hydrochlorothiazide (T-HCTZ), demonstrated diuretic use resulted a significantly decreased number of vertigo attacks, although it did not alter the progression of hearing loss, tinnitus or the degenerative progression of Meniere’s disease^8^.

The mechanism of sodium chloride (NaCl) triggering endolymphatic hydrops has been ascribed to the aberrant regulation of sodium by either the sodium–potassium adenosine triphosphatase (Na/K-ATPase) or the epithelial sodium channel (ENaC), both of which are expressed in the cochlea and provide sodium homeostasis^9–14^. Increased dietary salt consumption increases substances that are specific inhibitors and ligands of the Na/K-ATPase which might result in changes in the activity of the Na–K-ATPase in cochlea^15^. A low salt diet in a rat model increased Na/K-ATPase levels in stria vascularis which is responsible for secreting endolymph^16^. Animals fed compounds that inhibited ATPase together with a high salt diet had smaller endolymphatic sacs than animals fed a high salt diet without inhibiting the ATPase, thereby ascribing the volume of the endolymphatic sac to the activity of the ATPase^17^. Glucocorticoid receptors stimulate absorption of sodium^18^, as well as upregulation of ENaC^11^ and therefore may explain why some MD patients respond to corticosteroids.

High dietary NaCl consumption can contribute to variety of health conditions, including autoimmune disease. High dietary NaCl intake is linked with a higher probability of development and worsening of other autoimmune diseases such as systemic lupus erythematosus, rheumatoid arthritis. and inflammatory bowel disease^19^. Higher sodium intake is associated with increased clinical and radiological disease activity in patients with multiple sclerosis (MS)^20^. Mechanistically, increased salt consumption has been linked to aberrations in both the innate and adaptive immune responses. Innate immune system mechanism can be influenced by intake of excess NaCl^21^. Skin interstitial macrophages alter local electrolyte composition in response to NaCl-mediated extracellular hypertonicity and their regulatory role provides a buffering mechanism for salt-responsive hypertension in rats^21^. High-salt diets can induce Th17 development, and exacerbate disease in the experimental autoimmune encephalomyelitis (EAE) murine model of multiple sclerosis^22,23^. Increased in NaCl concentration triggers (serum-and glucocorticoid-inducible protein kinase-1) SGK1 expression which is mediator of sodium homeostasis^24,25^. Increased NaCl concentration also results in impaired Foxp3^+^ regulatory T cell function in human and murine in vitro and in vivo^26^, thus shifting the balance towards autoimmunity. A clinical study done by Yi et al. in 2014 showed that the healthy humans who consumed a high-salt diet were observed to have increased number of PBMCs and increased production of IL-6 and IL-23 compared with the healthy subjects on a low-salt diet^27^.

Six percent of unilateral MD and sixteen percent of bilateral MD have been hypothesized to have an autoimmune etiology^28^. Restriction of dietary salt, diuretics and use of corticosteroids are a few of the clinical management options^29^, although steroids are less effective in MD compared to AIED^30^. Patients with Autoimmune inner ear disease (AIED) have bilateral fluctuating sensorineural hearing loss, and up to 50% of episodes may have concomitant vertigo^31^. AIED patient are initially corticosteroid responsive in 70% of cases, however the response is lost over time^32,33^, although the mechanism of action of corticosteroid-resistance has not been fully elucidated^34^_._ Patients with bilateral Meniere’s disease appear clinically similar to AIED patients but may be distinguished based on the presence of vertigo at every attack in MD as compared with occasional vertigo during every attack of hearing loss in AIED, although clearly overlap appears to exist. It has been shown that 6.5% of cochlear hydrops patients were bilateral initially, and 26% progressed to bilateral hydrops, with one third converting to clinical Meniere’s disease^35^. Expression of pro-inflammatory cytokines, specifically IL-1, have been shown in MD patients^36^. Our previous studies have demonstrated elevated interleukin-1β (IL-1β) levels in corticosteroid-unresponsive autoimmune inner ear disease (AIED) patients^37^. We have identified that patients with AIED uniquely process IL-1β to a pro-inflammatory 28 kDa form as a result of caspase-7–mediated cleavage. This 28 kDa isoform of IL-1β is biologically active and strongly proinflammatory in response to LPS^38^. Although the pro-inflammatory properties of IL-1β have been ascribed to the canonical 17 kDa product of IL-1β^39^, we have observed that the 28 kDa product has almost equal ability to instigate downstream inflammation^38^. Given the role of NaCl in autoimmune disease, in the present study, we characterized the effect of NaCl on cellular pro-inflammatory cytokine release in PBMC from patients with classical MD, and whether diuretics or anakinra can ameliorate pro-inflammatory cytokine production in these patients.

## Results

We determined effects of NaCl in the ability to induce pro-inflammatory cytokine production in PBMC from a small cohort of definite MD patients and control subjects. Patient demographics of those included in these studies are shown in Table 1. Notably, controls recruited for these studies reported no personal or family history of MD, vertigo, hearing loss or autoimmune disease. All MD patients had active disease at the time of recruitment. A subset of MD patients was given corticosteroids at the time of recruitment for a decline in hearing (Table 1). The majority of MD patients did not respond (Table 1). Concomitant vestibular migraine was observed in 3 of the 14 patients with MD (Table 1).

**Table 1:** Clinical features of patients with MD.

Age/gender	Meniere’s disease	Degree hearing loss	Concomitant autoimmune disease?	Family hx MD	Medications	Steroids given immediately post-recruitment?	Steroid response	ConcomitantMigraine?
65M Caucasian Hispanic	Definite	Unilateral severe, flat	No	No	T-HCTZ	Yes	Yes	No
50M Caucasian	Definite	Unilateral, upsloping	No	No	T-HCTZ	Yes	No	No
76F Caucasian	Definite	Asymmetric, flat in affected ear (no hx/sx MD in contra ear, HF loss)	No	Yes	Previously T-HCTZ, at time of recruitment, none	No	N/A	No
61M Caucasian	Definite	Asymmetric, flat in affected ear (no hx/sx MD in contra ear, HF loss)	Psoriatic arthiritis	No	T-HCTZ	No	N/A	Yes
60F Caucasian	Definite	Unilateral upsloping	No	No	Betahistine	No	N/A	No
59F Caucasian	Definite	Unilateral, severe, flat	No	No	T-HCTZ	Yes	No	No
46F Caucasian	Definite	Unilateral, upsloping	No	Yes	Betahistine	No	N/A	No
50F Caucasian	Definite	Unilateral severe, flat	No	No	T-HCTZ Betahistine	Yes	No	No
56M Caucasian	Definite	Unilateral, severe, slight upslope	No	No	Acetazolamide Betahistine	Yes	No	No
65F Caucasian	Definite	Unilateral, upsloping	No	No	T-HCTZ, Betahistine	Yes	No* (previously responded)	No
56M Caucasian	Definite	Unilateral moderate, flat (previously upsloping)	No	No	Previously acetazolamide, at time of recruitment none	Yes	Yes	No
73M Caucasian	Definite	Unilateral, flat severe	No	No	T-HCTZ	No	N/A	No
46F Caucasian	Definite	Unilateral, flat severe	No	No	Unable to tolerate, hx endolymphatic shunt	Yes	Did not complete, unable to tolerate	Yes
39F Caucasian	Definite	Unilateral, severe, upsloping	No	No	Betahistine acetazolamide	Yes	No	Yes

Clinical data on 14 MD patients. Table includes gender, demographics, family history and medications.

### NaCl treatment results in dose-dependent increase in IL-1β and IL-6 m RNA levels in the PBMCs of MD patients

**Figure 1:**
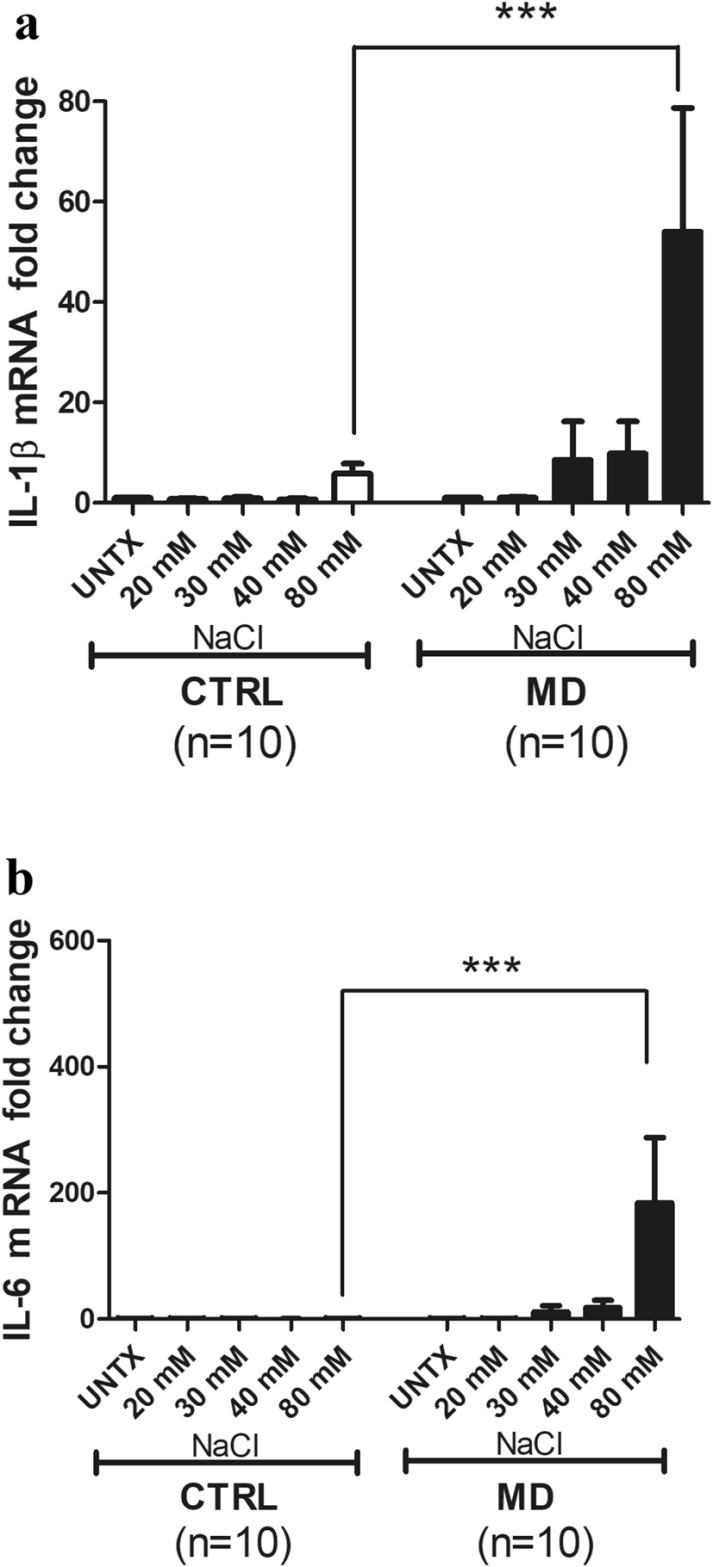
(**a**) NaCl treatment results in dose-dependent increase in IL-1β mRNA levels in the PMBCs of MD patients. PBMCs from MD patients (n = 10) and control subjects (n = 10) were treated with NaCl at the concentrations of 20, 30, 40 and 80 mM, LPS as positive control and compared with no treatment. The IL-1β expression levels were measured by Q-RT–PCR. Statistical analysis by one way ANOVA resulted in p = 0.0005. Applying a Bonferroni’s multiple comparison test still indicated a significant effect (p < 0.05) for comparison between 80 mM NaCl treatment in controls vs. 80 mM NaCl treatment in MD patients. (**b**) NaCl treatment results in dose-dependent increase in IL-6 mRNA levels in the PMBCs of MD patients. PBMCs from MD patients (n = 10) and control subjects (n = 10) were treated with NaCl at the concentrations of 20, 30, 40 and 80 mM, LPS as positive control along with no treatment. The IL-6 expression levels were measured by Q-RT–PCR. Statistical analysis by one way ANOVA resulted in p = 0.0035. Applying a Bonferroni’s multiple comparison test still indicated a significant effect (p < 0.05) for comparison between 80 mM NaCl treatment in controls vs. 80 mM NaCl treatment in MD patients.

We determined the dose dependent effect of NaCl stimulation in PBMC by Quantitative Real-Time PCR (Q-RT-PCR) from MD patients (n = 10) and control subjects (n = 10). A dose-dependent increase in IL-1β and IL-6 mRNA levels were observed in MD patients with maximal expression observed at 80 mM NaCl (Fig. 1a,b). In controls, NaCl stimulation of PBMC resulted in minimal mRNA expression of IL-1β and IL-6 (Fig. 1a,b). Statistical analysis by one-way ANOVA resulted in p = 0.0005 and p = 0.0035. For IL-1β and IL-6 respectively. Applying a Bonferroni’s multiple comparison test still indicated a significant effect (p < 0.05) for comparison between 80 mM NaCl treatment in controls vs. 80 mM NaCl treatment in MD patients for both IL-1β and IL-6 (Fig. 1a,b).

### NaCl treatment results in a dose-dependent increase in IL-1β and IL-6 release in the PBMCs of MD patients

**Figure 2:**
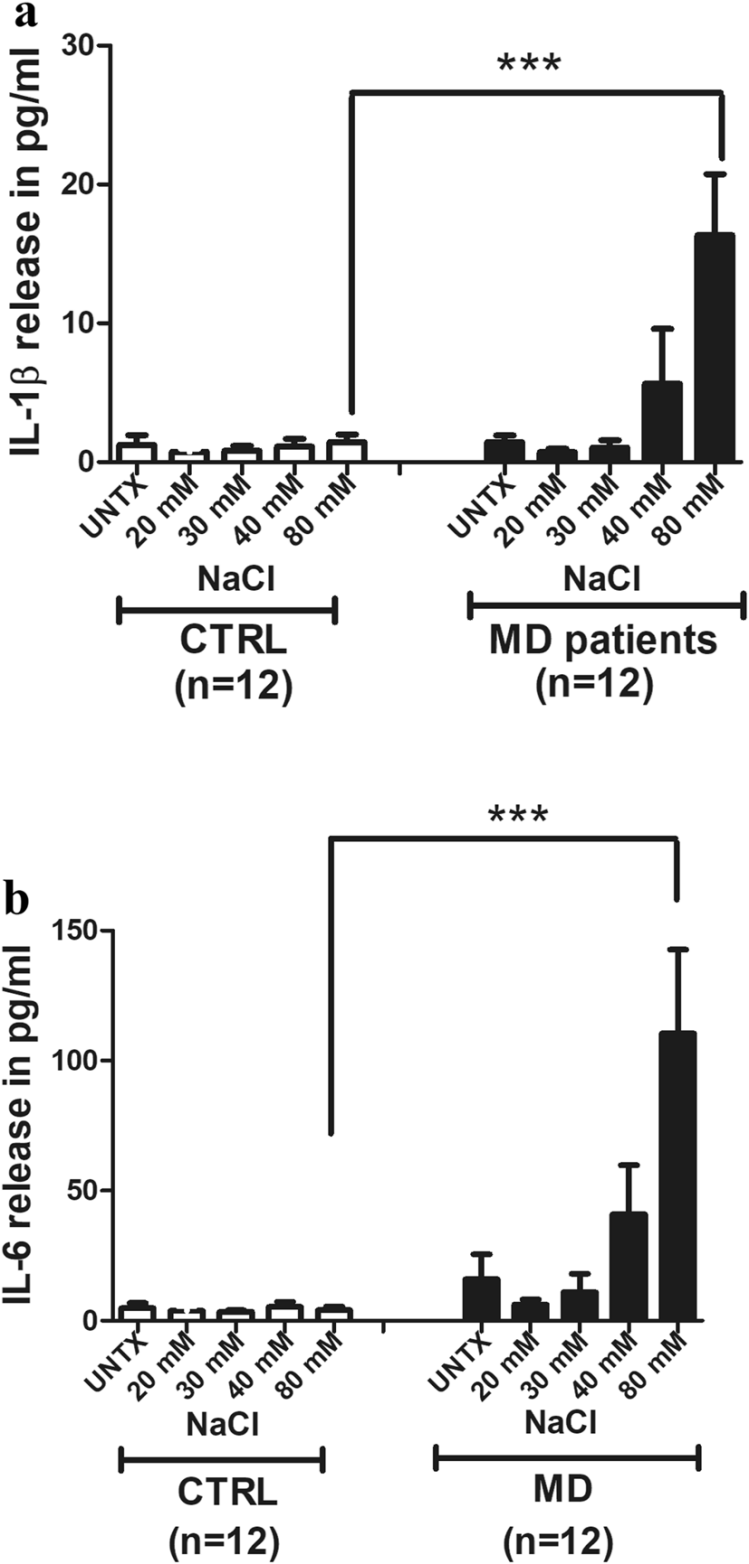
(**a**) NaCl treatment results in dose-dependent increase in IL-1β release in the PMBCs of MD patients. PBMCs from MD patients (n = 12) and control subjects (n = 12) were treated with either LPS (positive control), NaCl at the concentrations of 20, 30, 40 and 80 mM or left untreated. Supernatants were collected from the conditioned medium of MD patients and control subjects. IL-1β release was determined by ELISA. The bars show the mean protein release in pg/ml with ± SEM. Statistical analysis by one way ANOVA resulted in p = 0.0001. Applying a Bonferroni’s multiple comparison test still indicated a significant effect (p < 0.05) for comparison between 80 mM NaCl treatment in controls vs. 80 mM NaCl treatment in MD patients. (**b**) NaCl treatment results in dose-dependent increase in IL-6 release in the PMBCs of MD patients. PBMCs isolated from MD patients (n = 12) and control subjects (n = 12) were cultured over a 16 h period in the presence of increasing doses of sodium chloride, LPS (as positive control) or left untreated. Supernatants were collected from the conditioned medium. IL-6 release was determined by ELISA. The bars show the mean protein release in pg/ml with ± SEM. Statistical analysis by one-way ANOVA resulted in p = 0.0001. Applying a Bonferroni’s multiple comparison test still indicated a significant effect (p < 0.05) for comparison between 80 mM NaCl treatment in controls vs. 80 mM NaCl treatment in MD patients.

To determine whether the transcriptional induction of IL-1β and IL-6 resulted in increased circulating pro-inflammatory cytokines in the presence of sodium chloride, we performed a sandwich ELISA on MD patients (n = 12) and controls (n = 12). NaCl stimulation of PBMC again demonstrated a dose-dependent increase in IL-1β and IL-6 release in MD patients, (Fig. 2a,b). Statistical analysis by one-way ANOVA resulted in p = 0.0001 and p = 0.0001 for IL-1β and IL-6 respectively. Applying a Bonferroni’s multiple comparison test still indicated a significant effect (p < 0.05) for comparison between 80 mM NaCl treatment in controls vs. 80 mM NaCl treatment in MD patients for both IL-1β and IL-6 (Fig. 2a,b).

### NaCl treatment results in a dose-dependent increase in generation of 28 kDa IL-1β fragment in MD patients and control subjects fail to generate 28 kDa band of IL-1β

**Figure 3:**
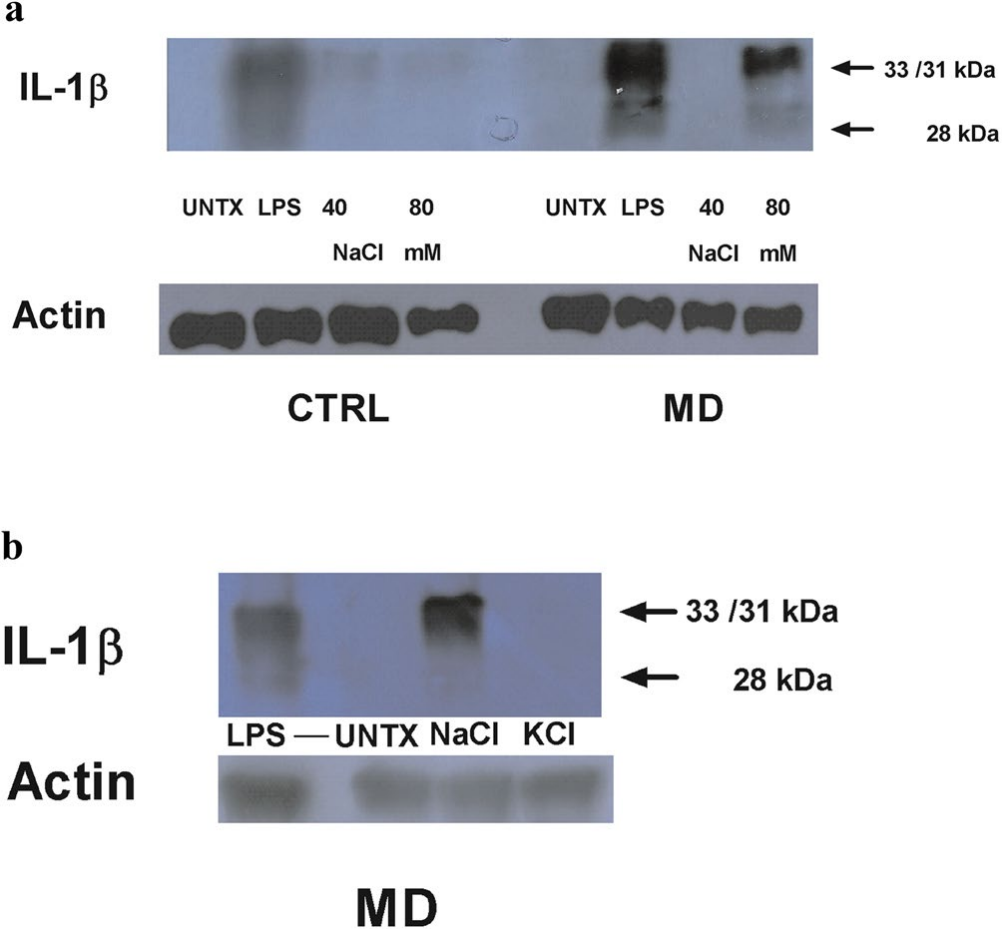
(**a**) NaCl treatment results increase in generation of 28 kDa IL-1β fragment in MD patients when compared to control subjects. PBMCs of control subject and MD patient were treated with NaCl at concentrations of 40 and 80 mM, LPS as positive control along with no treatment. The samples were subjected to Western blotting using IL-1β antibody. β-actin was used as internal control for immunoblotting. The representative blot for one of five MD patients and one of five control subjects are shown in the figure. (**b**) Potassium chloride failed to induce 28 kDa band of IL-1β in MD patients. PBMCs from MD patient were treated with Potassium chloride (KCl) (80 mM), NaCl (80 mM) along with LPS as positive control and kept untreated. The samples were subjected to Western blotting using IL-1β antibody. β-actin was used as a control for total protein.

When PBMCs isolated from MD patients were stimulated with NaCl and protein isolated for Western blot analysis, the 28 kDa fragment of IL-1β was observed in a dose-dependent manner in response to increasing NaCl in MD patients (n = 5) when compared to normal healthy subjects (n = 5). A representative blot shown in Fig. 3a. Notably, Potassium chloride (KCl) had no effect on IL-1 β induction, arguing that the observed induction of IL-1 β in response to NaCl is specific and cannot be ascribed to all salts (Fig. 3b)*.*

### Triamterene-hydrochlorothiazide (T-HCTZ) inhibited the NaCl-induced 28 kDa band of IL-1β in MD patients

**Figure 4:**
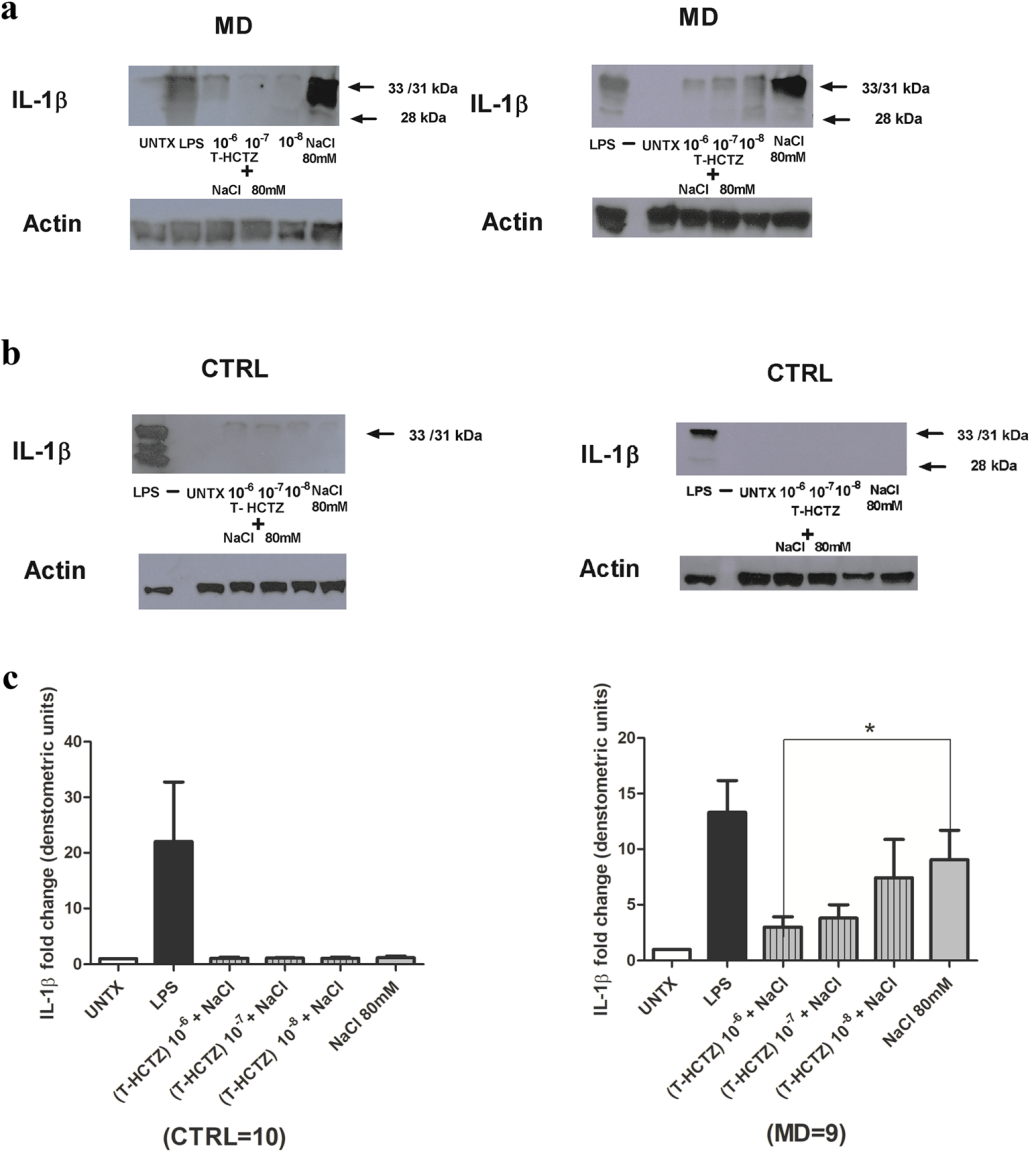
(**a**) T-HCTZ inhibited NaCl induced 28 kDa band of IL*-*1β in a dose-dependent manner in MD patients. PBMCs from MD patient were treated with increasing concentration of T-HCTZ (10^–6^ M to 10^–8^ M) in combination with NaCl at concentration 80 mM, along with LPS as positive control and kept untreated. The samples were subjected to Western blotting using IL-1β antibody. β-actin was used as a control for total protein. The representative blots for two out of nine MD patients are shown in the figure. (**b**) NaCl failed to induce IL-1β in control subjects. PBMCs from healthy controls were treated with increasing concentration of T-HCTZ in combination with NaCl at concentration 80 mM, along with LPS as positive control and kept untreated. The samples were subjected to Western blotting using IL-1β antibody. β-actin was used as a control for total protein. The representative blots for two out of ten control subjects are shown in the figure. (**c**) NaCl failed to induce IL-1β in control subjects. The histogram shows the quantitative densitometry of 28 kDa protein of IL-1β (fold over control) normalized over actin expression from ten control subjects and nine different MD patients. The data is shown as mean ± SEM.)*.* Statistical analysis by one-way ANOVA resulted in p = 0.0011. Applying a Bonferroni’s multiple comparison test still indicated a significant effect (p < 0.05) for comparison of T-HCTZ (10^−6^ M) and NaCl with NaCl alone.

Triamterene-hydrochlorothiazide (T-HCTZ) inhibited the observed NaCl induction of the 28 kDa band of IL-1β in a dose dependent manner in MD patients. PBMCs were treated with increasing concentrations of T-HCTZ (10^–8^ to 10^–6^ M) in combination with 80 mM NaCl. T-HCTZ at the concentration of 10^–6^ M was found to be most effective in mitigating the NaCl effect (Fig. 4a,b), the blots are representative of two of the nine blots performed)*.* Statistical analysis by one way ANOVA resulted in p = 0.0011. Applying a Bonferroni’s multiple comparison test still indicated a significant effect (p < 0.05) for comparison of T-HCTZ (10^−6^ M) and NaCl with NaCl alone (Fig. 4c).

### Anakinra inhibited NaCl induced 28 kDa band of IL-1β in MD patients

**Figure 5:**
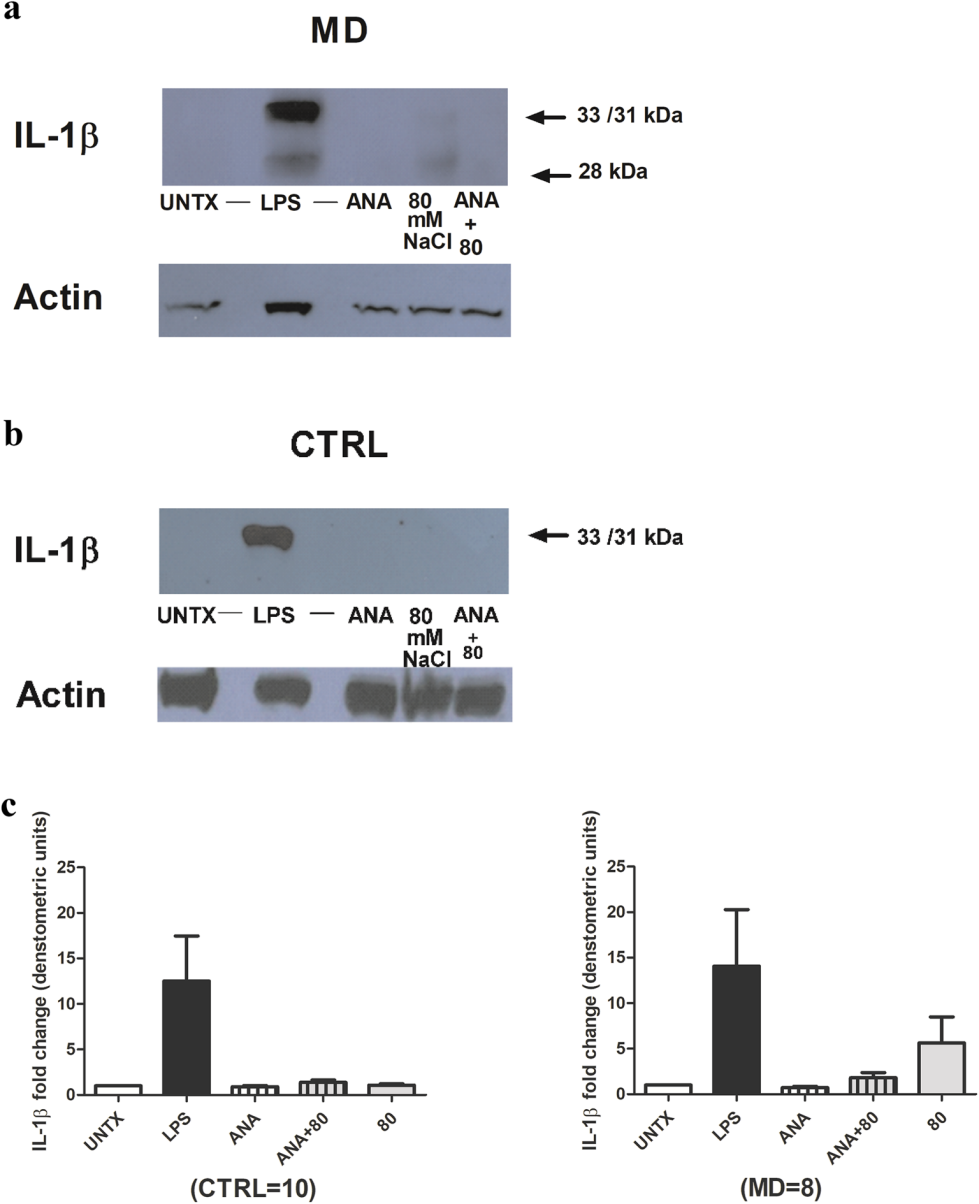
(**a**) Anakinra inhibited NaCl induced 28 kDa band of IL*-*1β in MD patients*.* PBMCs from MD patients (n = 8) were treated with anakinra 100 ng/ml with or without 80 mM NaCl, along with LPS as positive control and kept untreated. The samples were subjected to Western blotting using IL-1β antibody. β-actin was used as a control for total protein. (**b**) NaCl failed to induce 28 kDa band of IL-1β in control subjects and therefore effect of anakinra on NaCl induction could not accessed*.* PBMCs from control subjects (n = 10) were treated with anakinra 100 ng/ml alone or in combination with NaCl at concentration 80 mM, 80 mM salt alone, LPS as positive control and kept untreated. The samples were subjected to Western blotting using IL-1β antibody. β-actin was used as a control for total protein. The figure represents one blot out of 10 blots. (**c**) The 28 kDa band of IL-1β/actin ratio from the densitometry analysis of the Western blot bands from ten control subjects and eight different MD patients. The data is shown as mean ± SEM.

PBMCs of MD patients and control subjects were treated either with anakinra alone (at the concentration of 100 ng/ml), 80 mM NaCl alone or in combination and compared with LPS-stimulated and untreated. Anakinra was able to partially block sodium chloride-induced 28 kDa band generation in MD patients (Fig. 5, the blot is representative of one of 8 patients)*.*

## Discussion

We identified that high dietary NaCl diet triggers inflammation through the production and release of the pro-inflammatory cytokines IL-1β and IL-6 from PBMC, resulting in the clinical exacerbation of MD. The induction of pro-inflammatory cytokine transcription, translation and release was optimal at 80 mM of NaCl and specific, as KCl did not similarly induce IL-1β. The 40–80 mM concentration of NaCl has been used across many studies^40,41^, and is believed to represent the physiological concentration range of NaCl in the interstitium and lymphoid tissue^21,42^. In a study done by Shapiro and Dinarello^41^, a range of 40 to 80 mM NaCl was optimal for induction of another proinflammatory cytokine, IL-8, in PBMCs of healthy donors. We further demonstrated that the PBMCs of MD patients respond differently to dietary NaCl than the PBMCs of normal healthy controls: specifically, MD patients process IL-1β to the pro-inflammatory 28 kDa form in a dose-dependent manner in response to NaCl.

Vestibular migraine (VM) may co-exist with MD or it may mimic MD, as clinical symptoms overlap. Recent studies have demonstrated that these two entities can be distinguished by their cytokine profile, where vestibular migraine patients express significantly less IL-1β than MD patients^43,44^. In our studies, out of 14 MD patients studied, only 3 patients had both VM and MD, whereas 11 had MD alone. The plasma and conditioned supernatant values of these two groups were similar: VM + MD was 1.2 pg/ml and 2.56 pg/ml whereas MD was 1 pg/ml and 3.51 pg/ml, although we did not examine patients with VM alone which explains why a substantial difference was not observed. All patients were recruited during a period of active disease flare. In 9 of 14 patients, steroids were given immediately following recruitment because of an observed hearing decline and consistent with previous reports, the majority did not respond to corticosteroids. We also examined whether there was a difference in NaCl induced IL-1β release between these patients with a hearing decline necessitating corticosteroid use and those that did not (Table 1). Our results indicated that a nominal 1.4-fold difference in IL-1β release in response to NaCl in patients that necessitated corticosteroid therapy.

Resident macrophages have been identified in the stria vascularis and endolymphatic sac^40,45^ which are functionally similar to monocytes and microglia. Blockade of sodium channels (especially Nav1.6) in microglia attenuated the release of LPS-stimulated IL-1β^46^. Macrophages monitor tissue osmolarity and induce inflammation through NLRP3^47^. A drop in intracellular potassium and to a lesser degree an increase in intracellular sodium could enhance the activity of NLRP3^47^. A high salt diet has been associated with ENaC-dependent NLRP3 activation in dendritic cells in a murine model of hypertension^48^. It has been shown in the respiratory tract and kidneys that pro-inflammatory cytokines down-regulates expression of ENaC and Na/K ATPase, and interferes with sodium transport^49^. The diuretic triamterene is a known inhibitor of the Na/K-ATPase in the kidney plasma membrane^10,50^ and of ENaC^51^ therefore may exert an effect on multiple Na/K-ATPases, including those in immune cells. To address this question, we isolated monocytes from a patient with autoimmune disease and stimulated with NaCl in the presence of either T-HCTZ or ouabain ((an inhibitor of the Na/K-ATPase) (Supplemental Fig. 1)). T-HCTZ inhibited generation of the 28 kDa IL-1 product suggesting ENaC is involved in the processing of IL-1, whereas ouabain did not. However, the combination of ouabain and T-HCTZ abrogated expression of both the 28kD and the pro-IL-1 bands (33/31 kDa), suggesting a synergistic effect on IL-1 expression. Future studies determining the relative contributing roles of ENaC and the Na/K-ATPase in both MD and AIED may provide interesting new therapeutic targets for intervention in these diseases.

To our knowledge, this is the first study investigating the effects and correlating the role of high dietary NaCl diet in triggering inflammation through the production and release of pro-inflammatory cytokines such as IL-1β and IL-6 and induction of 28 kDa IL-1 band in MD patients*.* Our results indicated that diuretic T-HCTZ was effective in inhibiting 28 kDa IL-1β band in PBMC of MD patients at concentration of 10^–6^ M. The observation that T-HCTZ inhibited NaCl-induced IL-1β expression may provide additional mechanistic information as to the observed clinical benefit of the use of this diuretic in MD. In cardiac disease, use of diuretics has been observed to modulate an effect on pro-inflammatory cytokine release^52,53^. It is still unknown, and worthy of further exploration, as to whether during a MD attack, whether sodium dietary load alone is sufficient to trigger IL-1β processing in susceptible patients and whether these patients exhibit differential sensitivity to diuretics.

## Materials and methods

### Patient recruitment

This study was approved by the Institutional Review Board (IRB) of Northwell Health System. Written informed consent was obtained from all subjects. The study involved 14 MD patients and 14 control subjects. The mean age of patient group is 57, whereas the mean age of the control group is 44. The female: male ratio was 1.3:1 and 1:1 for patients and control subjects respectively. All experiments were performed in accordance with relevant guidelines and regulations. Patient characteristics can be found in Table 1.

### Reagents

Triamterene-hydrochlorothiazide (T-HCTZ), were dissolved in 1 ml DMSO and stock concentration of combination was 1 molar. The combination was vortexed for 3–5 min to create a homogenous solution. An equal amount of DMSO (Sigma-Aldrich) was used in the unstimulated condition, and other conditions to correct for the DMSO effect. Lipopolysaccharide (LPS) (Sigma-Aldrich) was used at 1 µg/ml as a positive control. To rule out any role of endotoxin in the expression of proinflammatory cytokines in response to NaCl, we used endotoxin-free cell-culture grade NaCl (Sigma-Aldrich, catalogue number S5886-500G) throughout our studies. Anakinra was purchased from Swedish Orphan Biovitrum AB (Sobi™).

### Culture and stimulation of PBMC

Heparinized blood was obtained, and peripheral blood mononuclear cells (PBMC) were isolated by centrifugation on Ficoll/Paque gradient as describe before^37^. PBMCs were cultured at 37 °C with 5% CO_2_ in standard RPMI 1640 medium (Thermo Fisher Scientific) supplemented with 10% heat-inactivated fetal bovine serum (Atlanta Biologicals), and 1% penicillin/streptomycin. in a 24-well plate (Costar) at concentration of 1 × 10^6^/ml. For every treatment, cells were kept for 16 h of incubation unless mentioned otherwise. Viability of cells was determined by a trypan blue dye exclusion test using Cellometer (Nexcelom Bioscience).

### Quantitative real time PCR (Q-RT-PCR)

PBMCs were harvested after completion of incubation for RNA isolation using RNeasy mini kit (Qiagen) as per the manufacturer’s protocol. All qPCR reactions were performed on ABI 7900HT Fast Real-time PCR System (Applied Biosystems) using qRT-PCR mastermix (Eurogentec) per the supplier’s instructions under the following cycling conditions: 30 min at 48 °C (1 cycle), 10 min at 95 °C (1 cycle), 15 s at 95 °C, and 1 min at 60 °C (40 cycles). Relative quantification was performed using GAPDH as an internal control. Fold changes for each gene were calculated using the ΔΔCT method. For GAPDH (Assay ID:Hs99999905_m1), and IL-6 (Assay ID:Hs00985639_m1) gene expression, pre-validated human TaqMan primer and probe sets were used. Since they are proprietary the sequences are not accessible (Thermo Fisher Scientific). IL-1β primer was designed with the Probe Library Assay Design Center. UPL probe number 78 was used for amplification of IL-1β. Primers used to measure the RNA expression of IL-1β were (forward) ctgtcctgcgtgttgaaaga (reverse) ttgggtaatttttgggatctaca.

### ELISA

IL-1β and IL-6 measurements were made in the cell supernatants, by using Quantikine Human IL-1β/IL-1F2 ELISA Kit and Human IL-6 Quantikine ELISA Kit (R&D Systems) following manufacturer’s instructions. Samples were quantified using corrected values of 450 and 570 nm, reading absorbance in a microplate reader (Thermo Fisher Scientific, accuSkan). A 4-parameter logistic curve was used to describe the data. For each experiment at least 2 replicates were used for analysis.

### Western blot

Cell lysates were prepared as previously described^25^. The protein content of the cell lysates was determined by bicinchoninic acid (BCA) method (Thermo Fisher Scientific). Protein (20 μg) per lane was electrophoresed through a 12% polyacrylamide gel (BIORAD) and electroblotted to a polyvinylidene difluoride (PVDF) membrane (BIORAD). Membranes were blocked with 5% non-fat dry milk (NFDM) (BIORAD) for 1 h, and then incubated with anti-IL-1β (R&D Systems) antibodies followed by horseradish peroxidase-conjugated mouse IgG (R&D Systems). Respective blots were also probed with anti-β-actin (clone AC-15, Millipore Sigma) antibody as loading controls. Enhanced chemiluminescence (Thermo Fisher Scientific) was used to visualize reactive protein bands on X-ray film. Densitometric analysis of Western blots was conducted using ImageJ software (National Institutes of Health).

### Statistical analysis

GraphPad Prism software version 5 was used to perform the statistical analysis. In all figures, standard error of the mean (SEM) are shown. Data groups were analyzed by one-way analysis of variance (ANOVA). Post hoc testing was performed using a Bonferroni’s Multiple Comparison Test. Statistical significance was achieved at p less than, or equal to 0.05.

## Supplementary Information

Supplementary Figure 1.

Supplementary Information.

## Abbreviations

AAO-HNS: American Academy of Otolaryngology–Head and Neck Surgery
AIED: Autoimmune inner ear disease
BCA: Bicinchoninic acid
DMSO: Dimethyl sulfoxide
EAE: Experimental autoimmune encephalomyelitis
ENaC: Epithelial sodium channel
IL: Interleukin
IRB: Institutional Review Board
KCl: Potassium chloride
KDa: Kilo Dalton
LPS: Lipopolysaccharide
MS: Multiple sclerosis
MD: Meniere’s disease
NaCl: Sodium chloride
Na^+^/K^+^-ATPase: Sodium–potassium adenosine triphosphatase
PBMC: Peripheral blood mononuclear cell
PVDF: Polyvinylidene difluoride
SGK1: Serum-and glucocorticoid-inducible protein kinase-1
T-HCTZ: Triamterene-hydrochlorothiazide
VM: Vestibular migraine
