# Supplementary material for: NaCl exposure results in increased expression and processing of IL-1β in Meniere’s disease patients
Source: Sci Rep. 2022 Mar 23;12:4957. doi: 10.1038/s41598-022-08967-7 (PMC8943007; doi:10.1038/s41598-022-08967-7)
Supplement: Supplementary file 1 — Supplementary Figure 1. [file 41598_2022_8967_MOESM1_ESM.docx]

**Supplemental figure 1**

**
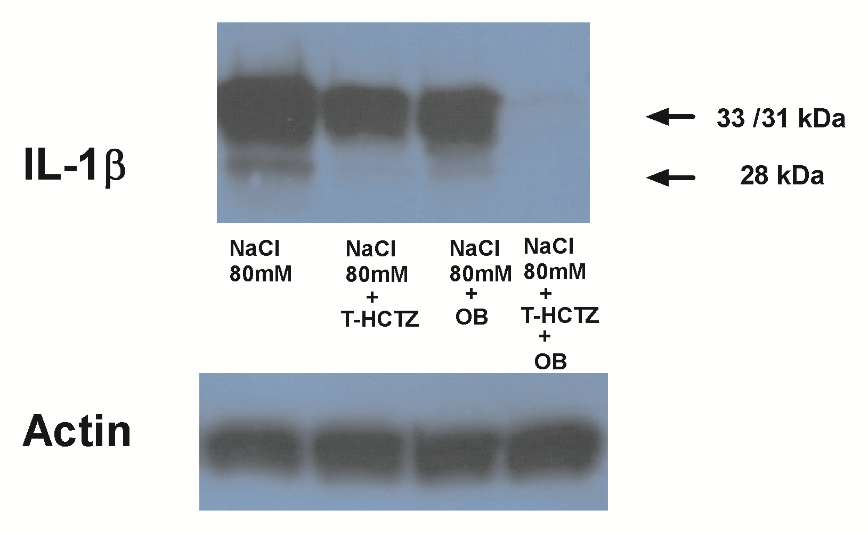
**

**Ouabain failed to inhibit NaCl induced 28kDa band of IL-1β**

Monocytes (isolated as mentioned previously)^56^ from an autoimmune patient were treated with either with 80mM NaCl, 80mM NaCl + T-HCTZ (10^-6^ M), 80mM NaCl +Ouabain (10^-7^ M) or 80mM NaCl + T-HCTZ + oubain. Protein was isolated and subjected to Western blotting using an anti-IL-1β antibody. β-actin was used as a control for total protein. Ouabain failed to inhibit the NaCl induced 28kDa band of IL-1β, whereas T-HCTZ partially inhibited the 28kDa band of IL-1β. The combination of T-HCTZ and ouabain completely inhibited 33/31 kDa band of IL-1β as well as the 28kDa band.
