# Supplementary material for: NaCl exposure results in increased expression and processing of IL-1β in Meniere’s disease patients
Source: Sci Rep. 2022 Mar 23;12:4957. doi: 10.1038/s41598-022-08967-7 (PMC8943007; doi:10.1038/s41598-022-08967-7)
Supplement: Supplementary file 2 — Supplementary Information. [file 41598_2022_8967_MOESM2_ESM.docx]

**Full unedited gel for figure 3a.**


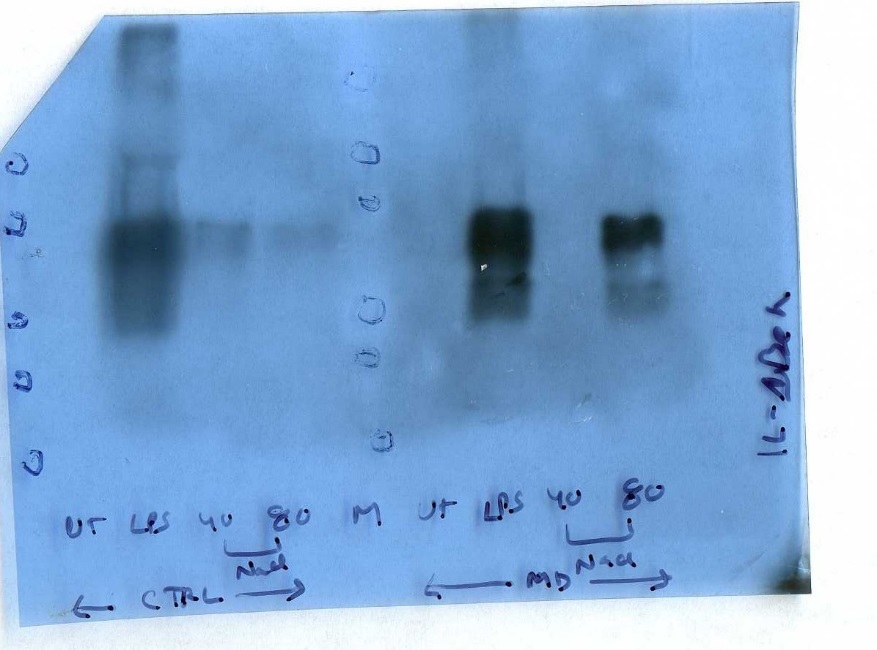


**Full unedited gel for figure-3a (IL-1β), none of the lanes were cropped.**


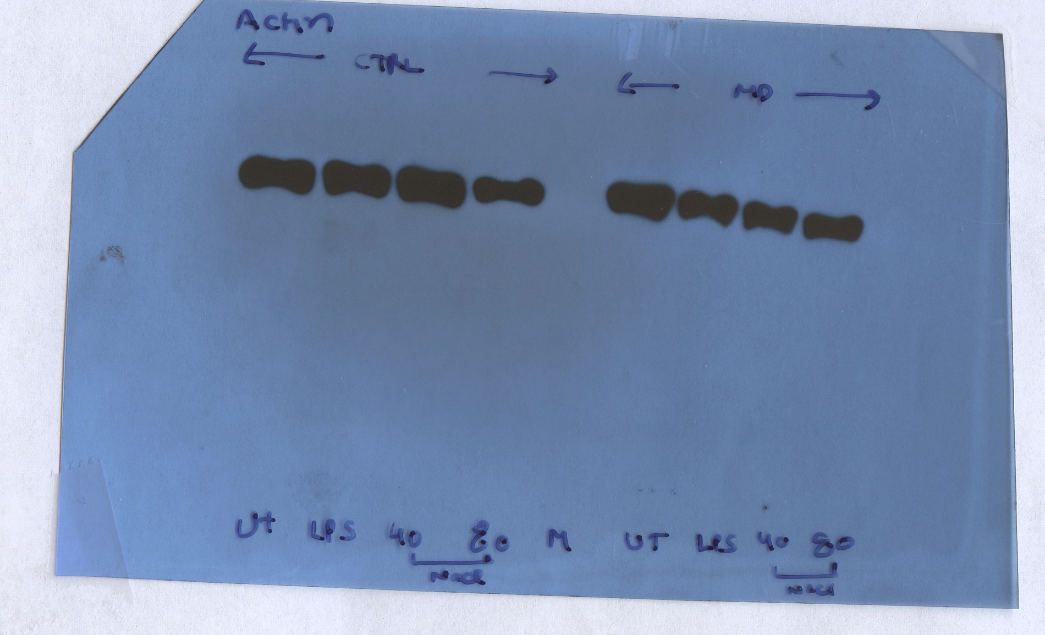


**Full unedited gel for figure-3a (actin), none of the lanes were cropped.**

**Full unedited gel for figure 3b. (MD)**


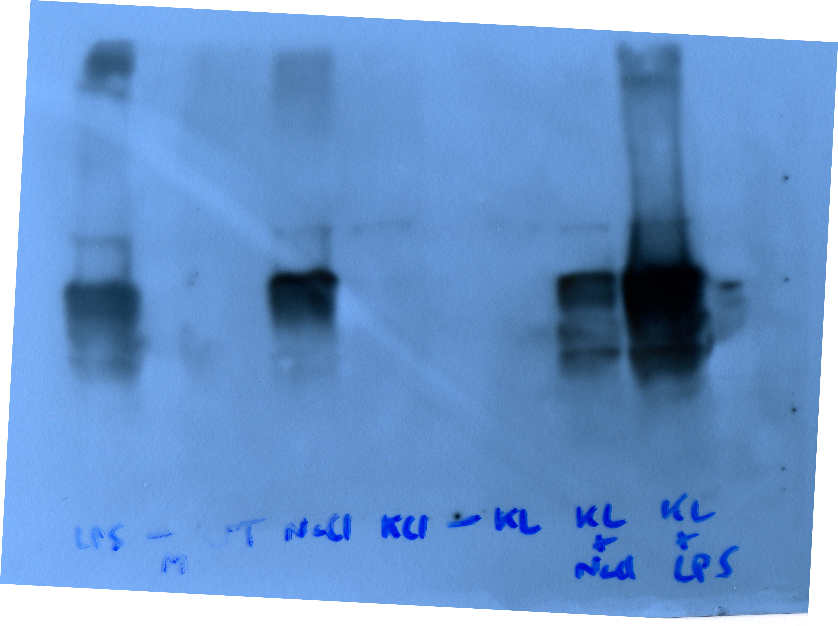


**Full unedited gel for figure3b (IL-1β), lanes 6 to 9 were cropped.**


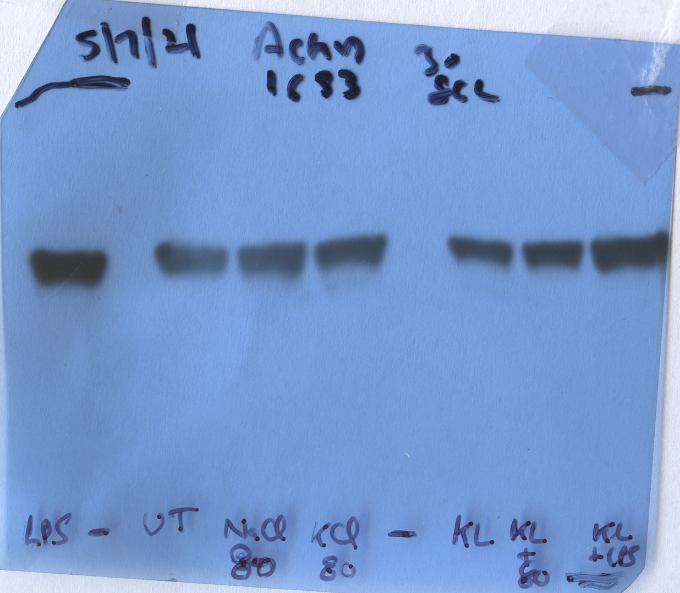


**Full unedited gel for figure3b (actin), lanes 6 to 9 were cropped.**

**Full unedited gel for figure 4a. MD#1**


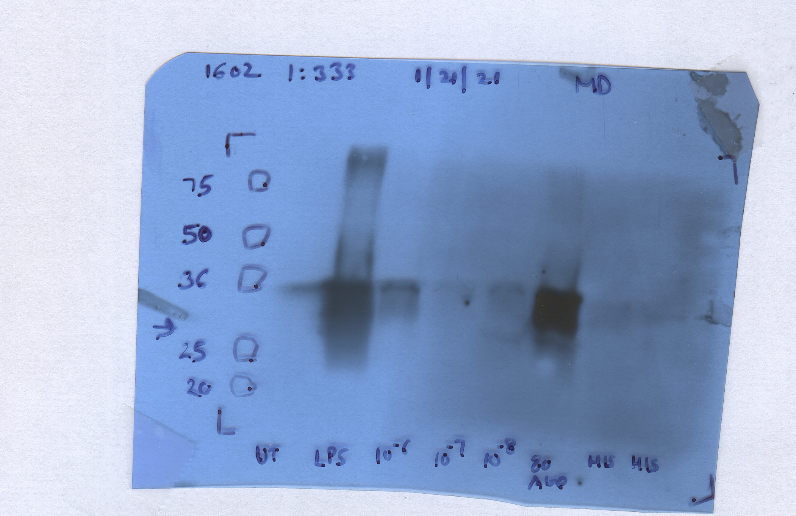


**Full unedited gel for figure 4a (IL-1β), lanes 7 and 8 were cropped.**


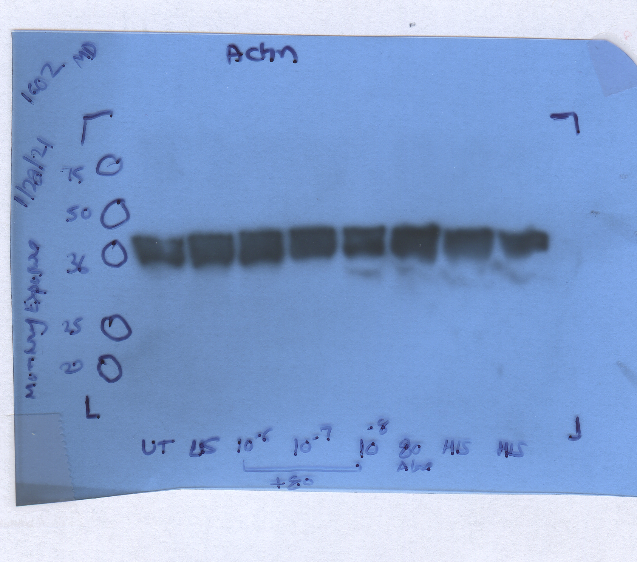


**Full unedited gel for figure 4a (actin), lanes 7 and 8 were cropped.**

**Full unedited gel for figure-4a. MD#2**


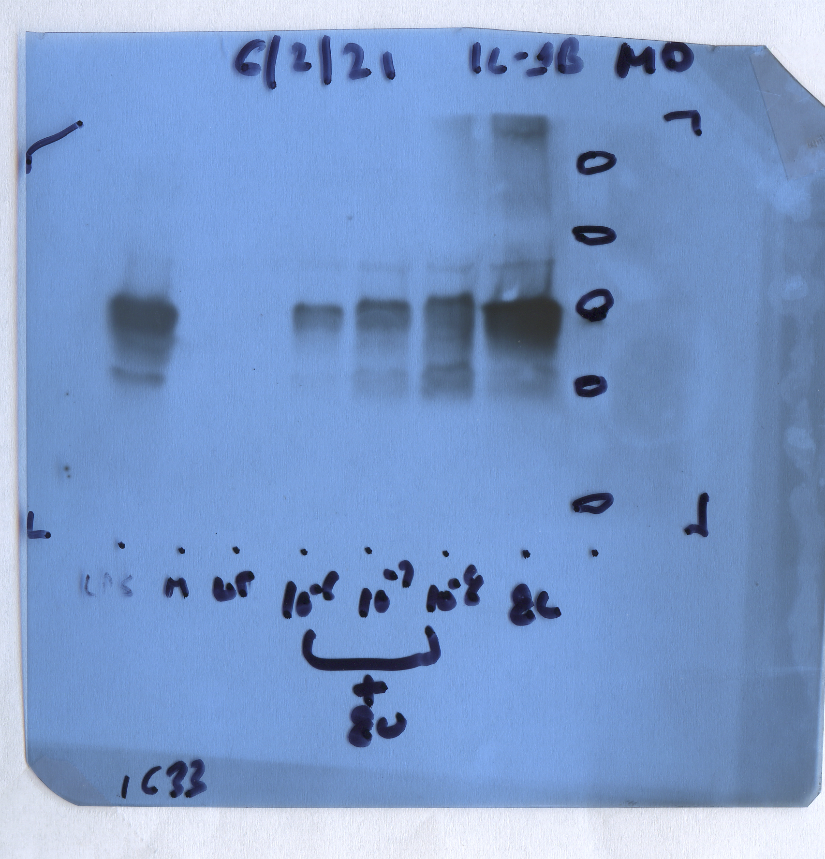


**Full unedited gel for figure-4a. (IL-1β), none of the lanes were cropped.**


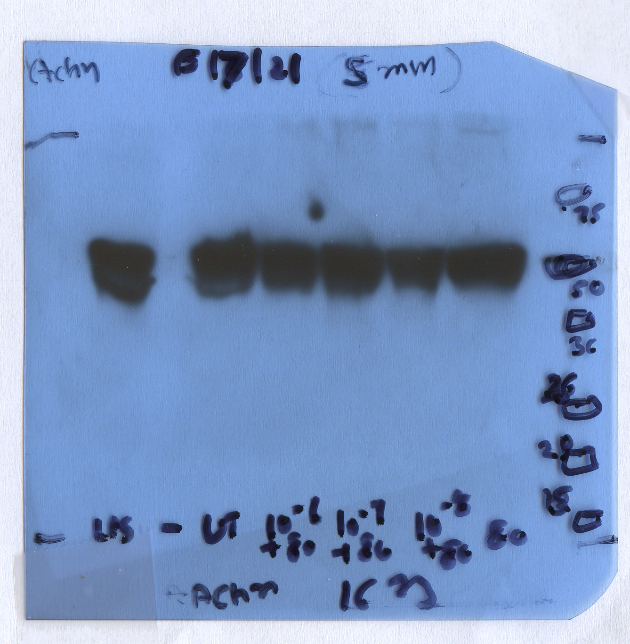


**Full unedited gel for figure-4a. (actin), none of the lanes were cropped.**

**Full unedited gel for figure-4b CTRL#1**

**
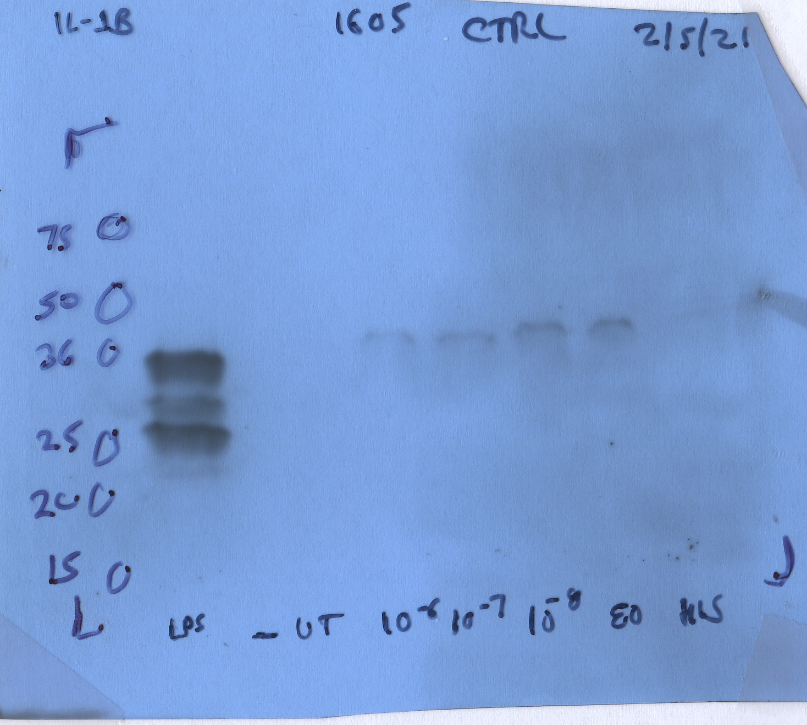
**

**Full unedited gel for figure-4b (IL-1β), lane 8 was cropped.**


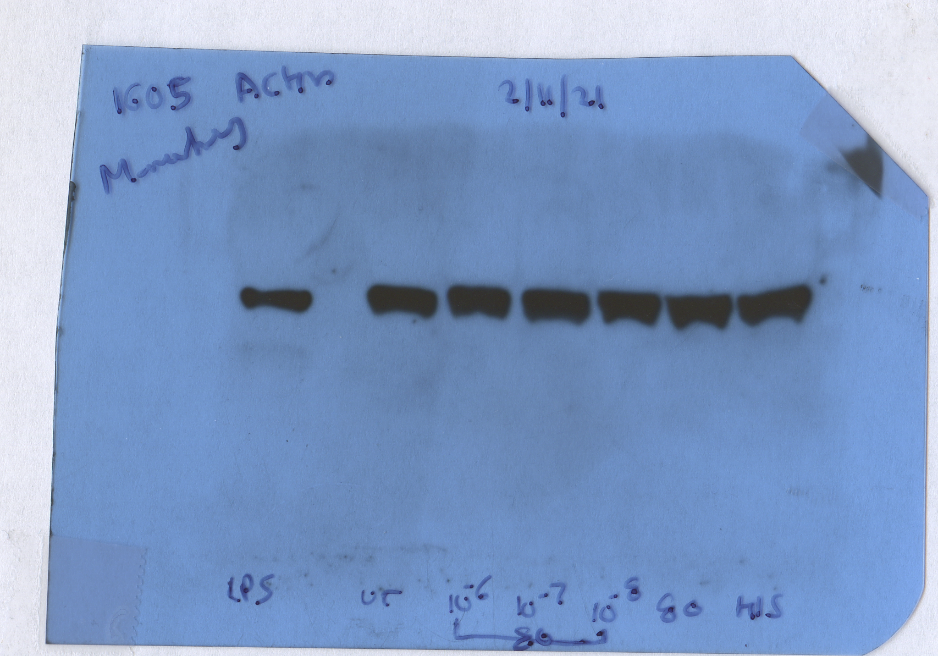


**Full unedited gel for figure-4b (actin), lane 8 was cropped.**

**Full unedited gel for figure-4b CTRL#2**


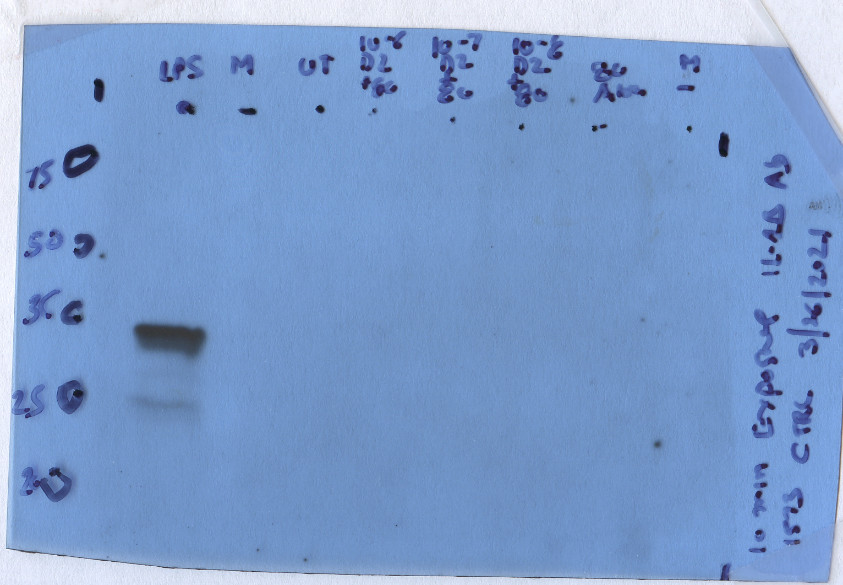


**Full unedited gel for figure 4b (IL-1β), none of the lanes were cropped.**


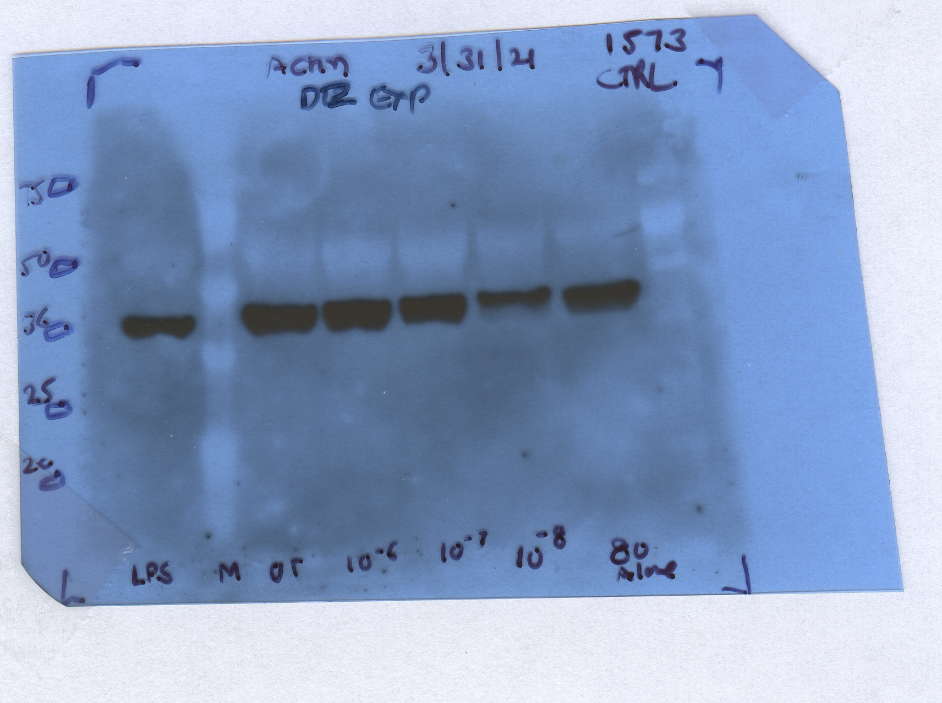


**Full unedited gel for figure 4b (actin), none of the lanes were cropped.**

**Full unedited gel for figure-5a (MD)**


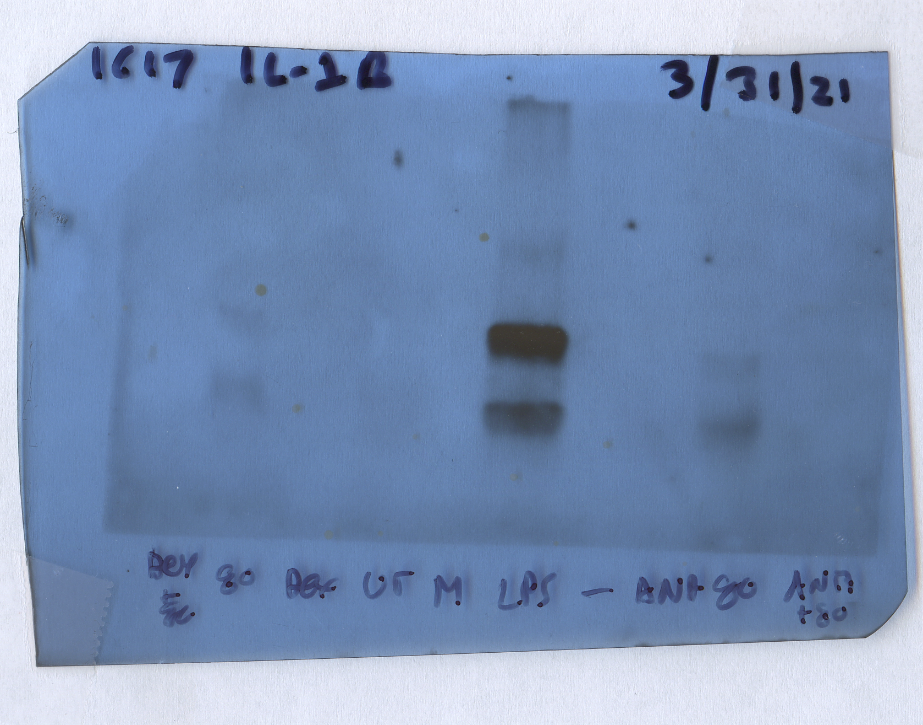


**Full unedited gel for figure 5a (IL-1β), lanes 1, 2 and 3 were cropped.**


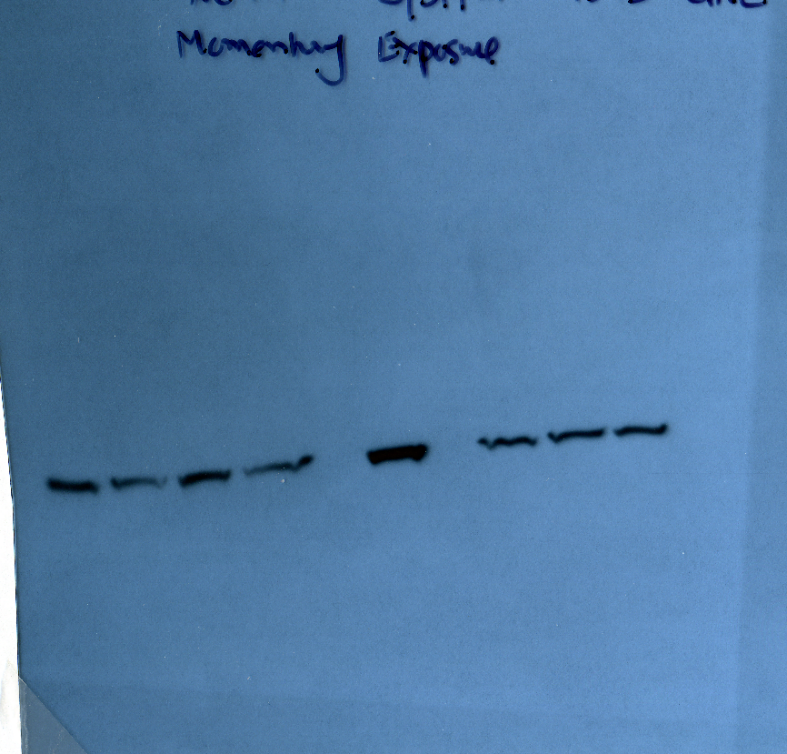


**Full unedited gel for figure 5a (IL-1β), lanes 1, 2 and 3 were cropped.**

**Full unedited gel for figure 5b (CTRL)**


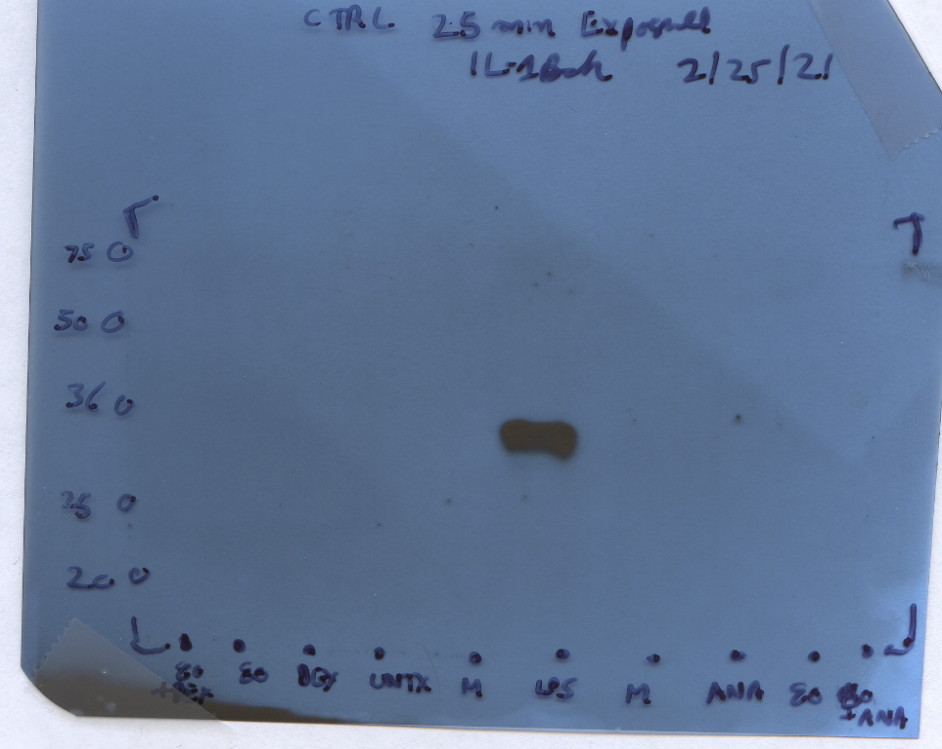


**Full unedited gel for figure 5b (IL-1β) lanes 1, 2 and 3 were cropped.**


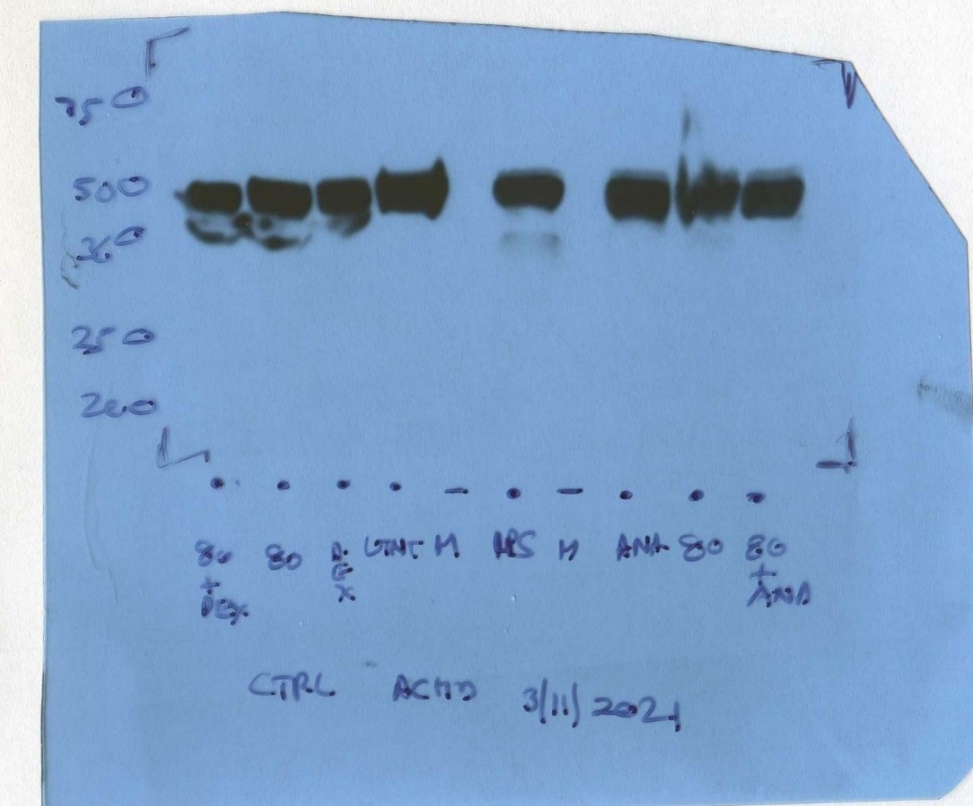


**Full unedited gel for figure 5b (actin) lanes 1, 2 and 3 were cropped.**
